# Vitamin D/vitamin D receptor protects intestinal barrier against colitis by positively regulating Notch pathway

**DOI:** 10.3389/fphar.2024.1421577

**Published:** 2024-07-26

**Authors:** Yanni Li, Yaoyu Guo, Chong Geng, Shuailing Song, Wenjuan Yang, Xiao Li, Chunhui Wang

**Affiliations:** ^1^ Department of Gastroenterology, West China Hospital of Sichuan University, Chengdu, China; ^2^ Laboratory of Gastroenterology and Hepatology, West China Hospital, Sichuan University, Chengdu, China

**Keywords:** tight junction, intestinal barrier, vitamin D, vitamin D receptor (VDR), ulcerative colitis, Notch pathway

## Abstract

**Objective:**

Vitamin D/Vitamin D receptor (VD/VDR) signaling and the Notch pathway are involved in intestinal barrier restoration in colitis; however, their relationship and underlying mechanism are largely unknown. Therefore, this study aimed to investigate the role and mechanism of VD/VDR and the Notch pathways in intestinal barrier protection.

**Methods:**

Genetic *Vdr* knockout (VDR KO) and VD deficient (VDd) mice were established, and colitis was induced by feeding 2.5% dextran sodium sulfate (DSS) water. Mechanistic studies, including real-time PCR, immunofluorescence, Western blotting and dual-luciferase reporter assays, were performed on cultured Caco-2 cells and intestinal organoids.

**Results:**

VD deficiency and VDR genetical KO increased the severity of DSS-induced colitis in mice, which presented a higher disease activity index score, increased intestinal permeability, and more severe intestinal histological damage than controls, accompanied by decreased and disrupted claudin-1 and claudin-3. Moreover, inhibition of Notch pathway by LY411,575 aggravated the severity of DSS-induced colitis and intestinal injury. In Caco-2 cells and intestinal organoids, the expression of Notch-1, N1ICD and Hes1 decreased upon downregulation or KO of VDR but increased upon paricalcitol (PAR, a VDR agonist) treatment. Meanwhile, PAR rescued claudin-1 and claudin-3 impairments that resulted from TNF-α exposure but failed to restore claudin-3 upon Notch inhibition. The dual-luciferase reporter assay further suggested that VD/VDR positively regulated the Notch signaling pathway by modulating Notch-1 transcription.

**Conclusion:**

VD/VDR positively modulates Notch activation by promoting Notch-1 transcription to maintain intestinal tight junction integrity and barrier function. This highlights the VD/VDR-Notch pathway as a potential new therapeutic target for protecting the intestinal barrier against ulcerative colitis.

## 1 Introduction

Ulcerative colitis (UC) is a type of inflammatory bowel disease that imposes a huge medical burden and affects the physical and mental health of patients (Agrawal and Jess, 2022). The pathogenesis of UC is complex and involves multiple factors such as the environment, genetics, intestinal infections, and inflammation. Intestinal barrier dysfunction is regarded as the predominant factor involved in the occurrence and development of the disease (Xavier and Podolsky, 2007). Mucosal healing has become increasingly important in the management of patients with UC. The intestinal mucosal barrier mainly consists of biological, chemical, mechanical, and immune barriers (Peterson and Artis, 2014; Xie et al., 2022). The mechanical barrier, which includes the intestinal epithelial cells and tight junctions (TJs) between cells and is regarded as the basic architecture of the intestinal barrier, determines the permeability of the intestinal barrier. An increase in intestinal permeability resulting from TJs impairment (e.g., claudin-1 protein downregulation and disruption) is associated with intestinal pathogen invasion and continuous immune activation, which plays critical roles in the development of UC (Martini et al., 2017). Therefore, intestinal barrier restoration is a promising therapeutic strategy for the treatment of UC.

Patients with UC are deficient in vitamin D (VD), a lipid-soluble vitamin, and this deficiency is associated with disease progression and relapse (Kabbani et al., 2016; Gubatan et al., 2017; Olmedo-Martín et al., 2019). VD exerts its function by binding to vitamin D receptor (VDR), a nuclear receptor, to regulate downstream molecules (Rochel, 2022). Various studies have indicated that VD/VDR signaling protects intestinal homeostasis through multiple mechanisms, such as maintaining the integrity of the intestinal barrier and regulating the immune system (Olmedo-Martín et al., 2019; Fakhoury et al., 2020; Sun and Zhang, 2022). VD/VDR signaling is not only directly regulates mature immune functions but also involved in immune cell differentiation (Ao et al., 2021). Besides, VD/VDR signaling has been found to modulate TJ proteins (e.g., claudins, occludin, and ZO-1) to maintain the integrity of the intestinal barrier (Pereira et al., 2011; Liu et al., 2017; Lee et al., 2021); however, the underlying molecular mechanism is not fully understood and requires further investigation.

The Notch pathway is essential for the maintenance of intestinal epithelial renewal and integrity (Dahan et al., 2011; VanDussen et al., 2012). It involves ligands (such as jagged-1 and delta-like 1), receptors (such as Notch-1), and downstream target genes in mammals (Siebel and Lendahl, 2017). Notch signaling is activated by ligand binding to receptors, leading to a series of enzymatic digestion reactions that release the Notch intracellular domain (NICD) (Vinson et al., 2016; Siebel and Lendahl, 2017). After translocating into the nucleus, NICD binds to transcription factors to promote the transcription of target genes (such as *Hes1*) (Vinson et al., 2016). Previous studies have reported that inhibition of the Notch pathway could result in spontaneous colitis and TJ impairment in mice, indicating a regulatory role of Notch signaling in maintaining the intestinal TJ barrier (Dahan et al., 2011; Obata et al., 2012; Liu et al., 2018). Although a previous report suggested that VD might mediate the Notch pathway (Qiu et al., 2021), the underlying mechanism remains unclear.

The present study aimed to investigate the role and mechanism of VD/VDR and the Notch pathways in intestinal barrier protection. Through various *in vivo* and *in vitro* experiments, we report that VD/VDR modulates Notch activation to protect and restore TJ integrity and intestinal barrier function in colitis.

## 2 Materials and methods

### 2.1 Animals

Genetic *Vdr* knockout (VDR KO) and wild-type (WT) littermates were bred from *Vdr* heterozygotes (B6.129S4-Vdr^tm1Mbd^/J; constructed by Jackson Laboratory) on a C57BL/6 background, and VDR deletion was confirmed as previously reported (Guo et al., 2023a; Guo et al., 2023a). Normal female C57BL/6 mice were purchased from Charles River Laboratories (Beijing, China) to maintain the fertility of *Vdr* heterozygotes. Other female and male mice for colitis were also purchased from Charles River Laboratories. All mice were maintained in specific pathogen-free conditions under a 12-h light cycle at an appropriate temperature (18°C–23°C) and humidity (40%–60%). To construct a VD-deficient (VDd) model, weaned mice were fed the VDd solid diet for 12 weeks and were sustained until sacrifice. VDR KO mice received a rescue diet (20% lactose, 2% calcium, and 1.25% phosphorus) after 4 weeks of age. All diets were designed by Research Diets, Inc. (NJ, United States), and the formulas for the diets are listed in Table 1.

**TABLE 1:** Diet’s formula for mice (gm).

Ingredient	Control diet	VD deficient diet	VDR KO rescue diet
Casein	200	200	200
DL-Methionine	3	3	3
Sucrose	100	100	300
Corn Starch	397.486	397.486	150
Soybean oil	70	70	—
Cellulose	50	50	50
Vitamin Mix^a^	10.0 (V10037)	10.0 (V13203)	10.0 (V10001)
Mineral Mix	35.0	35.0	35.0
Maltodextrin	132	132	—
Tert-butylhydroquinone	0.014	0.014	—
Choline bitartrate	2.5	2.5	2.0
Corn Oil	—	—	50
Lactose	—	—	200
Calcium Phosphate, dibasic	—	—	37.3
Calcium Carbonate	—	—	9.58
Total	1,000	1,000	1,046.88

^a^ V13203 is lack of vitamin D3. V10037 and V10001 contain 100,000 IU/gm vitamin D3.

### 2.2 Animal experiments

Colitis was induced in mice (with gender matching) by providing them *ad libitum* access to drinking water containing 2.5% dextran sodium sulfate (DSS; 9011–18-1; MP Biomedicals, Santa Ana, CA, United States) for 5 days, followed by normal drinking water for 2 days (details in Supplementary Figure S1). Disease activity index (DAI) was assessed based on weight loss, fecal characteristics, and hematochezia, as reported previously (Li et al., 2021). For Notch inhibition, the mice were gavaged with 10 mg/kg LY411,575 (S2714; Selleck, Shanghai, China), a γ-secretase inhibitor, in the last 4 days (once per day). For VDR activation, the mice were intraperitoneally administered 300 ng/kg/day paricalcitol (PAR; S6681; Selleck) 7 days before DSS drinking water was provided and were sustained until the last day. Intestinal permeability was assessed by measuring the concentration of serum fluorescein isothiocyanate–dextran (FD-4; 60842-46-8; Sigma-Aldrich, Shanghai, China), as previously reported (Li et al., 2021). Following anesthesia with isoflurane (VETEASY; Shenzhen, China), the mouse eyeballs were removed at the last day to collect blood samples in a dark environment. Serum samples were obtained via centrifugation at 3000 rpm for 15 min and stored in light-protected tubes at −80°C for future use. Distal colonic tissue was carefully isolated and immediately fixed in 4% paraformaldehyde or stored at −80°C for subsequent use.

### 2.3 Cell culture and experiments

Caco-2 cells were cultured in Dulbecco’s modified Eagle’s medium (DMEM; Gibco, United States) containing 10% fetal bovine serum (Biological Industries, China) in a humidified incubator with 5% CO_2_ at 37°C. All experiments were performed over 4–15 passages at 60%–80% confluency. For siRNA transfection, the cells were cultured in a 12-well plate and were transfected with 10 μM targeted siRNA or control RNA (provided by RIBOBio, China) using jetPRIME transfection kit (Polyplus, Bioparc, France) as previously reported (Guo et al., 2023b). PAR was used for VDR activation. The Caco-2 cells were incubated in complete medium with 100 nM PAR for 24 h. For Notch inhibition, LY411,575 (1 μM) was added into the medium for 28 h (4 h before PAR intervention). To mimic inflammatory stimulation, 100 ng/mL TNF-α (300-01A; PeproTech, Rocky Hill, NJ, United States) was added to cultured Caco-2 cells for 18 h (6 h after PAR pretreatment).

### 2.4 Intestinal organoids culture

Intestinal organoids were cultured from the extracted intestinal crypt cells of mice, as previously reported (Sugimoto and Sato, 2017; Mizutani and Clevers, 2020). Briefly, a 10 cm jejunum adjacent to the stomach was removed, washed with ice-cold Dulbecco’s phosphate-buffered saline (D-PBS), and cut into pieces. The segments were rinsed with ice-cold D-PBS 15 times and incubated in Gentle Cell Dissociation Reagent (07174; STEMCELL Technologies, Shanghai, China) for 20 min at room temperature. Dissociated intestinal crypts were filtered through 70 mm strainers and resuspended in DMEM/F12 medium containing 15 mM HEPES (H1095; Solarbio, Beijing, China). After calculating the number, the dissociated intestinal crypts were resuspended in IntestiCult^TM^ organoid growth medium (STEMCELL Technologies) and Matrigel (356,230; Corning, Shanghai, China) at a 1:1 ratio. After 10 min, organoid growth medium was added to the plate. The IntestiCult^TM^ organoid growth medium was replaced every other day, and the intestinal organoids were cultured and passaged every 7 days. The cultured organoids were harvested for immunofluorescence (IF) staining, Western blotting, and quantitative real-time PCR.

### 2.5 Histopathological evaluation

Distal colonic sections (4 μm) were stained with hematoxylin-eosin (H&E) for histological assessment. Histological scores were determined blindly by two independent investigators based on previously described criteria: severity of inflammation (0–2), lesion depth (0–3), crypt destruction (0–4), and lesion extent (0–4) (Guo et al., 2023b).

### 2.6 IF staining

Paraffin-embedded sections (distal colonic tissue and cultured organoids) were deparaffinized with xylene and rehydrated using gradient concentration alcohol (100%–95%–90%–85%–0%). Antigen retrieval was performed in boiling citrate buffer (pH 6.0; BL604A; Biosharp, Hefei, China) in a pressure cooker for 15 min, followed by natural cooling to room temperature. The sections were blocked in 5% goat serum (SL038; Solarbio) at 37°C for 1 h and were then incubated overnight with anti-claudin-1 (1:100; YT0942; Immunoway, TX, United States), anti-claudin-3 (1:100; 34–1700; Invitrogen, Shanghai, China), anti-cleaved Notch-1 (1:100; YC0067; N1ICD, Immunoway), and anti-Hes1 (1:200; GTX108356; GeneTex, Alton, IL, United States) antibodies in a humid environment at 4°C. The cultured cells were transferred onto coverslips, fixed in 4% paraformaldehyde, and permeabilized with 0.2% Triton X-100 (BioFroxx, Einhausen, Germany). After blocking with 5% goat serum for 1 h, the cells were incubated with the primary antibodies of interest (as described above). The samples were incubated with fluorescein isothiocyanate- or rhodamine-conjugated secondary antibodies (1:200; ab150081 and ab150114; Abcam, Cambridge, United Kingdom) for 2 h, followed by incubation with 4,6-diamidino-2-phenylindole (DAPI) staining solution (C1005; Beyotime Biotechnology, Shanghai, China) for 3 min. The samples were observed under a normal fluorescence microscope (DP72; OLYMPUS, Tokyo, Japan). Quantification for claudin-1 and claudin-3 were determined by the fluorescent integrated density (IntDensity) per high power field (HPF) using ImageJ software (Java1.8.0_172, NIH, United States) (Nathani et al., 2023). Quantification for Hes-1 and N1ICD were determined by the ratio of positive staining cells per crypt (Lopez et al., 2016). Quantified analysis for IF staining was made in 3–5 non-overlapping fields (about 10–15 crypts) to generate the average value for each mouse.

### 2.7 RNA isolation and quantitative real-time PCR assay

Total RNA was extracted from tissues and cells using the RNAiso Plus reagent (ForeGene, Chengdu, China) according to the manufacturer’s instructions. RNA (100 ng) was reverse-transcribed to cDNA using a reverse transcription mix kit (ForeGene). The mRNA expression of the target genes was detected via quantitative real-time PCR (qPCR) using a SYBR Green Master Mix kit (ForeGene). The expression levels of target genes were normalized to that of the endogenous control *Gapdh* using the ΔΔCT method. Primer sequences were listed in Table 2.

**TABLE 2:** Sequences of Primers for qPCR.

Gene	Species	Forward 5’–3’	Reverse 5’–3’
*Gapdh*	*M*	CAT​GGC​CTT​CCG​TGT​TCC​TA	CCT​GCT​TCA​CCA​CCT​TCT​TGA​T
*Jagged-1*	*M*	CCT​CGG​GTC​AGT​TTG​AGC​TG	CCT​TGA​GGC​ACA​CTT​TGA​AGT​A
*Jagged-2*	*M*	CAA​TGA​CAC​CAC​TCC​AGA​TGA​G	GGC​CAA​AGA​AGT​CGT​TGC​G
*Notch-1*	*M*	GAT​GGC​CTC​AAT​GGG​TAC​AAG	TCG​TTG​TTG​TTG​ATG​TCA​CAG​T
*Notch-2*	*M*	ATG​TGG​ACG​AGT​GTC​TGT​TGC	GGA​AGC​ATA​GGC​ACA​GTC​ATC
*Notch-3*	*M*	AGT​GCC​GAT​CTG​GTA​CAA​CTT	CAC​TAC​GGG​GTT​CTC​ACA​CA
*Notch-4*	*M*	CTC​TTG​CCA​CTC​AAT​TTC​CCT	TTG​CAG​AGT​TGG​GTA​TCC​CTG
*Dll-1*	*M*	CAG​GAC​CTT​CTT​TCG​CGT​ATG	AAG​GGG​AAT​CGG​ATG​GGG​TT
*Dll-4*	*M*	TTC​CAG​GCA​ACC​TTC​TCC​GA	ACT​GCC​GCT​ATT​CTT​GTC​CC
*Hes1*	*M*	TCT​GGA​GCT​GGT​GCT​GAT​AAC	CGG​TAG​CAC​TAT​TCC​AGG​ACC
*Hes5*	*M*	AGT​CCC​AAG​GAG​AAA​AAC​CGA	CGA​AGG​CTT​TGC​TGT​GTT​TCA
*Hey1*	*M*	AGC​GTG​GGA​AAG​GGA​TGG​TT	GAG​GAG​CTG​TAG​TCT​GGG​TG
*Heyl*	*M*	CAG​CCC​TTC​GCA​GAT​GCA​A	CCA​ATC​GTC​GCA​ATT​CAG​AAA​G
*Gapdh*	*H*	GTC​TCC​TCT​GAC​TTC​AAC​AGC​G	ACC​ACC​CTG​TTG​CTG​TAG​CCA​A
*Jagged-1*	*H*	GTC​CAT​GCA​GAA​CGT​GAA​CG	GCG​GGA​CTG​ATA​CTC​CTT​GA
*Jagged-2*	*H*	TGG​GCG​GCA​ACT​CCT​TCT​A	GCC​TCC​ACG​ATG​AGG​GTA​AA
*Notch-1*	*H*	GAG​GCG​TGG​CAG​ACT​ATG​C	CTT​GTA​CTC​CGT​CAG​CGT​GA
*Notch-2*	*H*	CCT​TCC​ACT​GTG​AGT​GTC​TGA	AGG​TAG​CAT​CAT​TCT​GGC​AGG
*Notch-3*	*H*	TGG​CGA​CCT​CAC​TTA​CGA​CT	CAC​TGG​CAG​TTA​TAG​GTG​TTG​AC
*Notch-4*	*H*	GAT​GGG​CTG​GAC​ACC​TAC​AC	CAC​ACG​CAG​TGA​AAG​CTA​CCA
*Dll-1*	*H*	GAC​GAA​CAC​TAC​TAC​GGA​GAG​G	AGC​CAG​GGT​TGC​ACA​CTT​T
*Dll-4*	*H*	TGG​GTC​AGA​ACT​GGT​TAT​TGG​A	GTC​ATT​GCG​CTT​CTT​GCA​CAG
*Hes1*	*H*	CCT​GTC​ATC​CCC​GTC​TAC​AC	CAC​ATG​GAG​TCC​GCC​GTA​A

M: *mus musculus*; H: *Homo sapiens*.

### 2.8 Western blot analysis

Total proteins were analyzed by Western blotting. After protein transfer, a polyvinylidene fluoride membrane was incubated with anti-N1ICD (110 kD; 1:1000; YC0067; Immunoway), anti-Hes1 (around 30 kD; 1:1000; GTX108356; GeneTex), anti-VDR (48 kD; 1:1000; ab109234; Abcam), anti-Notch-1 (around 125 kD; 1:1000; ET1606-55; HuaBio, Hangzhou, China), and anti-GAPDH (36 kD; 1:10^4^; YM3029; Immunoway) antibodies and blocked with 5% milk. The secondary antibody used was an anti-rabbit/mouse horseradish peroxidase-conjugated secondary antibody (1:10^4^; ZB2301/ZB2305; ZSGB Biotechnology, Beijing, China). The signal was detected using ECL Western blot detection reagent (P0018; Beyotime Biotechnology). Protein levels were presented as target protein intensities relative to those of GAPDH. Quantitative analysis of the intensity of each band was performed using the Quantity One imaging software (Bio-Rad Laboratories, Hercules, CA, United States).

### 2.9 Dual-luciferase reporter assay

To construct the dual-luciferase reporter system, the GV238 vector (MCS-firefly-luciferase) was selected and constructed to include the *Notch-1* promoter (Notch-1) or a negative control (CON). *VDR* cDNA and a vector (pcDNA3.1-CMV-3*flag; HANBIO, Shanghai, China) were linked and cloned using the HB infusion^TM^ system to construct a plasmid containing *VDR* cDNA (pVDR). Caco-2 cells cultured in a 12-well plate were transfected with plasmid (1 μg total DNA) using a jetPRIME transfection kit (Polyplus). Cellular lysis was detected using a Dual-Luciferase Reporter Assay Kit (HB-DLR; HANBIO) according to the manufacturer’s instructions. Firefly/luciferase fluorescence intensity was calculated to represent the intensity of *Notch-1* transcription.

### 2.10 Statistical analysis

Data are reported as the mean ± SEM. Student’s *t*-test was used to compare data between two groups. One-way analysis of variance was used to compare mean values among groups, followed by Tukey’s *post hoc* multiple comparison test. The repeated measures ANOVA was used to compare repeated measures with Bonferroni *post hoc* multiple comparisons. Statistical analyses were performed using GraphPad Prism 9.0 (GraphPad Software, San Diego, CA, United States). Statistical significance was set at *p*–value < 0.05.

## 3 Results

### 3.1 VD deficiency and VDR KO aggravate DSS-induced colitis accompanied by TJ impairment

**FIGURE 1:**
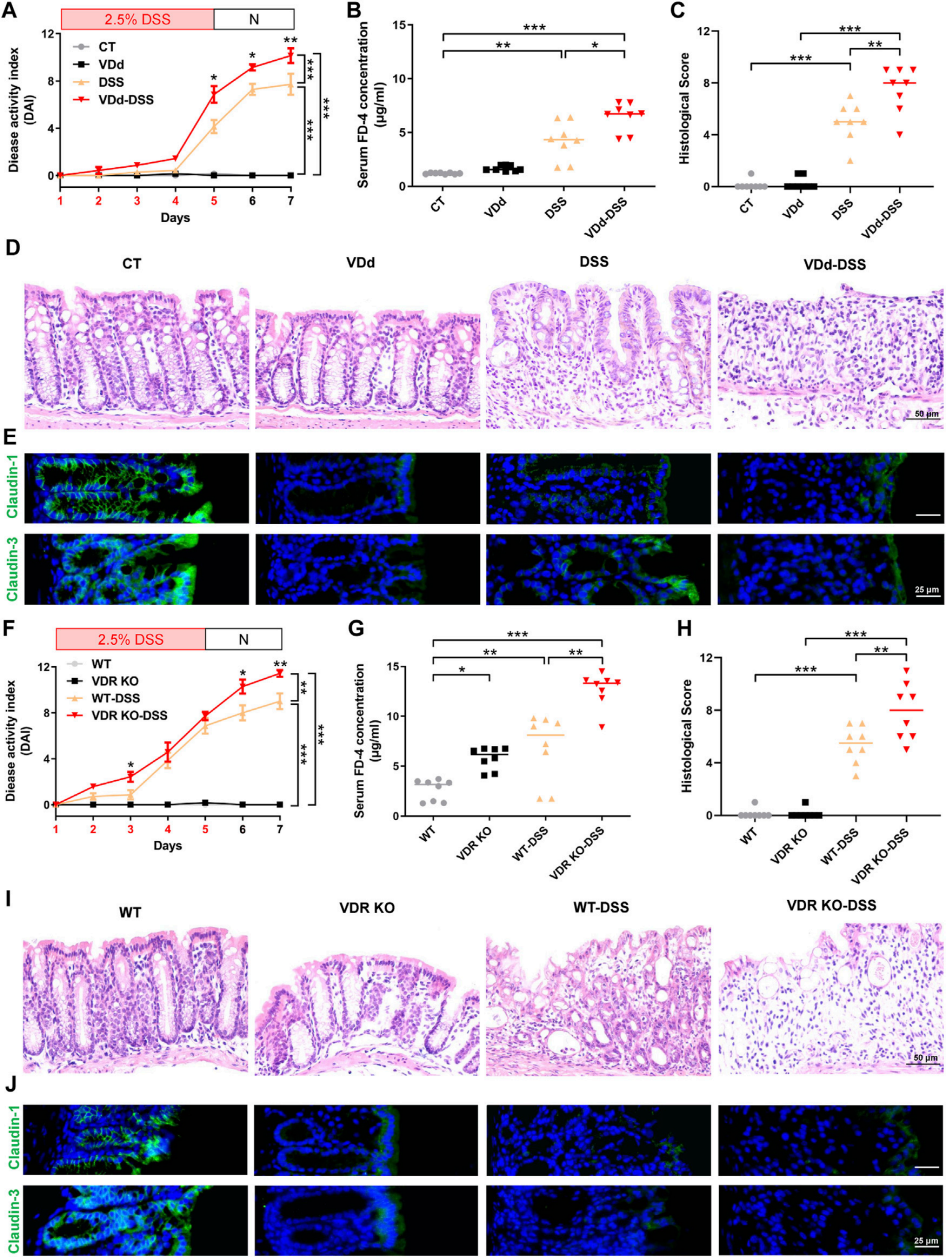
VD deficiency/VDR KO aggravates DSS-induced colitis. Vitamin D-deficient (VDd) and control (CT) feeding mice, and vitamin D receptor knockout (VDR KO) and wildtype WT littermates were induced colitis by feeding 2.5% DSS (days were marked as red in the *x*-axis) containing water for 5 days and normal water (N) for 2 days. **(A, F)** DAI scores of VDd and CT mice **(A)** and VDR KO and WT mice **(F)** with or without DSS feeding. DAI scores were compared by the repeated measures ANOVA with Bonferroni *post hoc* multiple comparisons. **(B, G)** Serum FD-4 concentration on each group. Statistical analysis was performed using ANOVA with Tukey’s post-test. **(C, H)** Histological scores of distal colons in each group. Statistical analysis was performed using ANOVA with Tukey’s post-test. **(D, I)** Representative colonic H&E staining image of each group (scale bar = 50 μm). **(E, J)** Representative colonic immunofluorescence (IF) staining image of claudin-1 (*green*), claudin-3 (*green*), and the nucleus (stained with DAPI, *blue*) (scale bar = 25 μm). Data are presented as the mean ± SEM from 8 mice in each study group. *: *p* < 0.05, **: *p* < 0.01, ***: *p* < 0.001.

To understand the effects of VD/VDR on the severity of colitis in mice, we first induced colitis in VDd mice by feeding them with DSS-containing water (VDd-DSS). Compared with those in the single DSS group, the DAI score in VDd-DSS group significantly increased in the last 3 days (Figure 1A) with the serum FD-4 concentration markedly increasing (Figure 1B). Colonic histological assessment showed VD deficiency aggravated DSS-induced colonic epithelium impairment (Figures 1C, D). Assessment of the TJ barrier showed that the expression and distribution of claudin-1 and claudin-3 were simultaneously disrupted (Figure 1E; Supplementary Figure S2A). Consistent trends in DAI scores and serum FD-4 concentration were also observed in VDR KO mice with colitis when compared to WT mice with DSS-induced colitis (Figures 1F, G). Moreover, colonic histological assessment indicated that VDR KO aggravated the severity of DSS-induced colonic epithelial impairment (Figures 1H, I) and claudin-1 and claudin-3 disruption (Figure 1J; Supplementary Figure S2B). Notably, under physiological conditions, we found an increased serum FD-4 concentration in VDR KO mice than in WT mice and disrupted expression and maldistribution of colonic claudin-1 and claudin-3 in VDd and VDR KO mice. Overall, VD/VDR signaling is critical for protecting the epithelial barrier against colitis.

### 3.2 Notch inhibition aggravates DSS-induced colitis and TJ impairment

**FIGURE 2:**
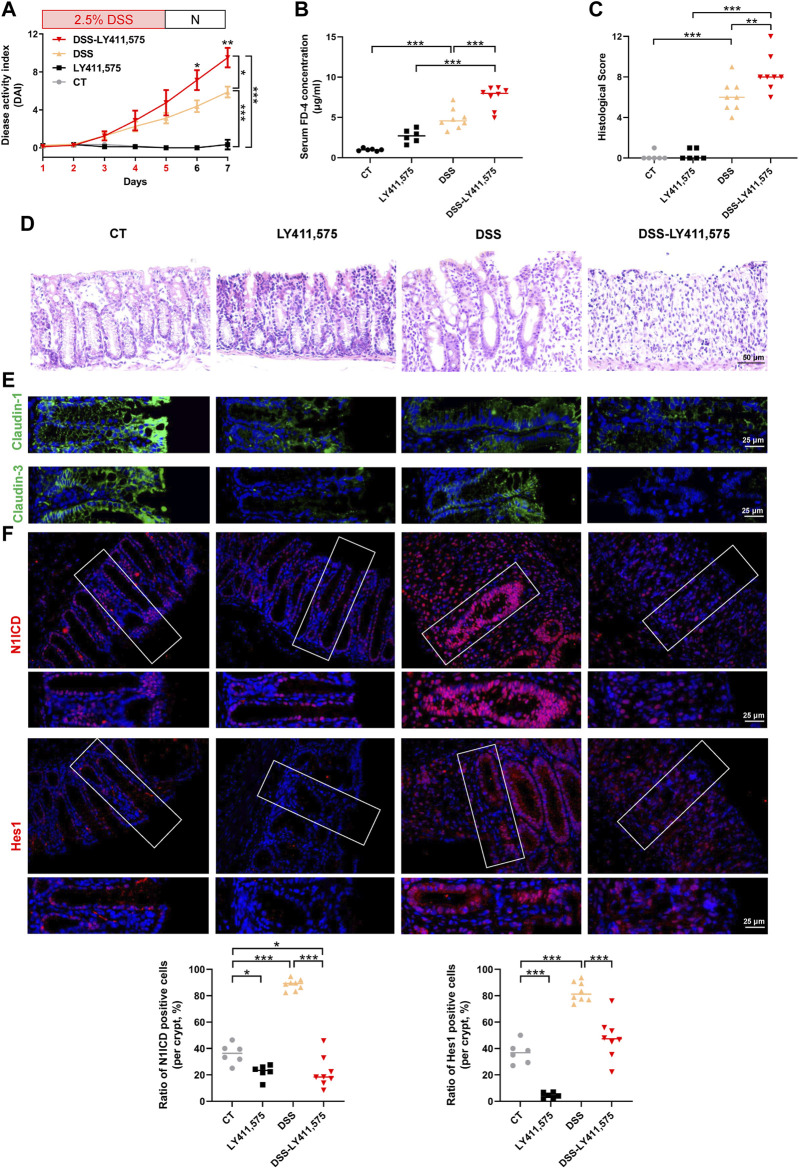
Inhibition of the Notch pathway aggravates DSS-induced colitis. CT: control, LY411,575 (Notch inhibitor): 10 mg/kg LY411,575 gavage, DSS: 2.5% DSS water, DSS-LY411,575: LY411,575 was intragastric administrated at the last 4 days of DSS feeding (once per day). **(A)** DAI scores of each group. DAI scores were compared by the repeated measures ANOVA with Bonferroni *post hoc* multiple comparisons. **(B)** Serum FD-4 concentration in each group. Statistical analysis was performed using ANOVA with Tukey’s post-test. **(C)** Histological scores of distal colons in each group. Statistical analysis was performed using ANOVA with Tukey’s post-test. **(D)** Representative colonic H&E staining image of each group (scale bar = 50 μm). **(E)** Representative colonic IF staining image of claudin-1 (*green*) and claudin-3 (*green*) (scale bar = 25 μm). **(F)** Representative colonic IF staining image and quantification of N1ICD (*red*) and Hes1 (*red*) (scale bar = 25 μm). Quantification of N1ICD and Hes1 were determined by the ratio of positive staining cells per crypt. Data are presented as the mean ± SEM from 6-8 mice in each study group. *: *p* < 0.05, **: *p* < 0.01, ***: *p* < 0.001.

To reveal the role of Notch signaling in intestinal epithelial barrier, we used the specific inhibitor LY411,575 in subsequent experiments. As shown in Figure 2A, LY411,575 gavage obviously increased DAI scores in DSS-treated mice. In addition, LY411,575 gavage increased the serum FD-4 concentration in DSS-induced colitis (Figure 2B). Colonic histological assessment confirmed that inhibition of Notch by LY411,575 aggravated DSS-induced colonic epithelium injury (Figures 2C, D) and claudin-1 and claudin-3 impairment (Figure 2E; Supplementary Figure S2C). Furthermore, IF staining verified the inhibitory effects of LY411,575, as evidenced by the suppression of N1ICD and Hes1. Interestingly, in 2.5% DSS-induced colitis mice, we found that both the fluorescence intensity and nuclear translocation of N1ICD and Hes1 increased, suggesting the activation of Notch signaling under DSS-treatment conditions (Figure 2F). Taken together, these results indicate that the Notch pathway is involved in intestinal barrier alternation and that inhibition of this pathway aggravates the severity of DSS-induced colitis and TJ impairment.

### 3.3 VD/VDR signaling regulates the Notch-1/Hes1 pathway and TJ

**FIGURE 3:**
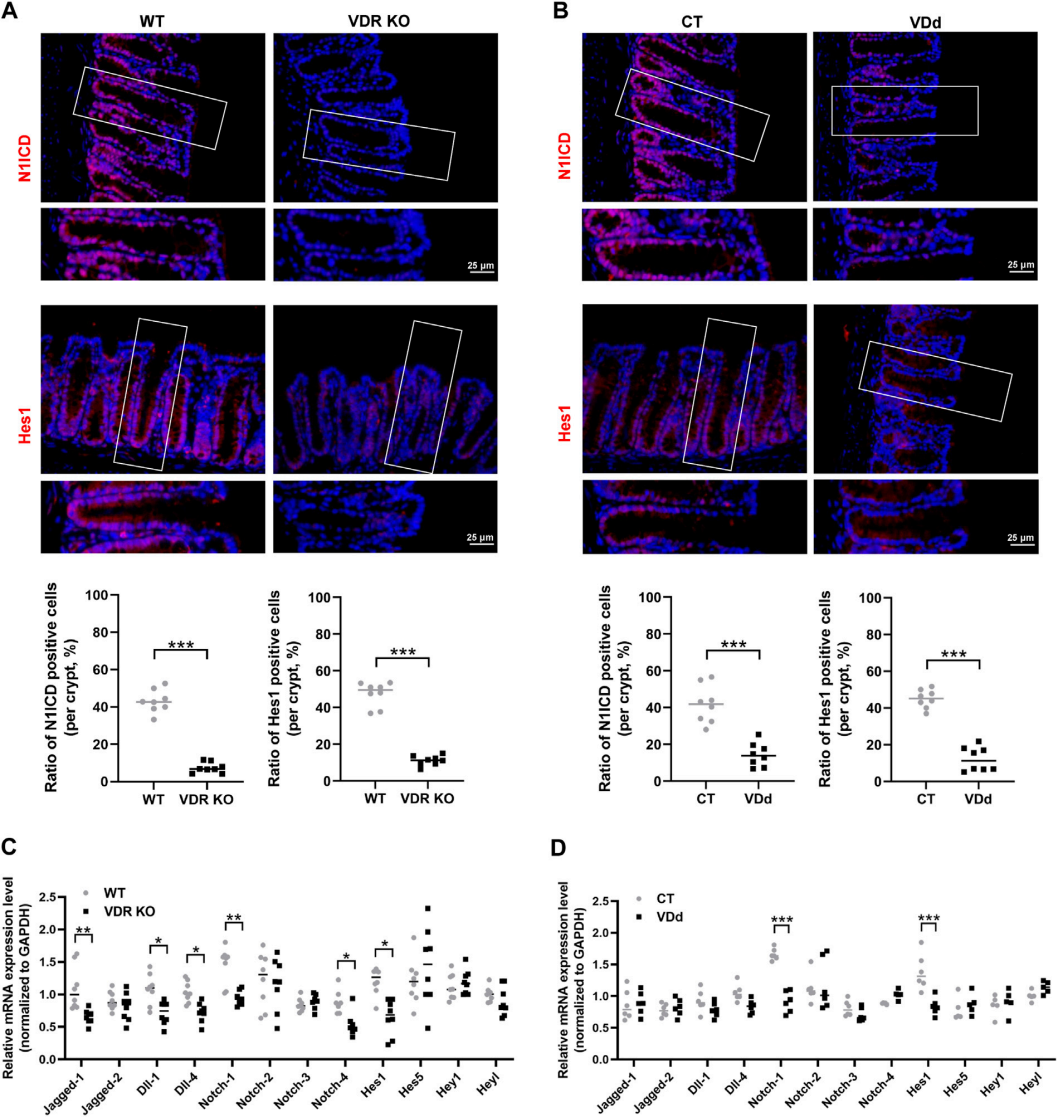
Notch pathway is inhibited in the colon under VDd or VDR KO conditions. **(A, B)** Representative colonic IF staining image and quantification of N1ICD (*red*) and Hes1 (*red*) in VDR KO **(A)** and VDd **(B)** mice (scale bar = 25 μm). **(C, D)** Real-time PCR analysis of the gene expression of Notch ligands, receptors, and effectors in the colon in VDR KO **(C)** and VDd **(D)** mice. Expression levels (VDR KO vs. WT or VDd vs. CT) were compared using paired *t*-test. Data are presented as the mean ± SEM from 6-8 mice in each study group. *: *p* < 0.05, **: *p* < 0.01, ***: *p* < 0.001.

Since VD/VDR signaling and the Notch pathway are both related to DSS-induced colitis and TJ integrity, we speculated that the Notch pathway might participate in the regulation of VD/VDR in TJ integrity. Therefore, we investigated the effects of VD/VDR signaling on the Notch pathway. Our results displayed that compared with that in WT mice, the Notch pathway in VDR KO mice was suppressed, as evidenced by a decrease in Hes1 and N1ICD expression under physiological conditions (Figure 3A). Similar trends were observed in VDd mice (Figure 3B). To elucidate the effect of VD/VDR signaling on the Notch pathway, we measured the expression of Notch-related ligands, receptors, and downstream effectors in the colons of VDR KO and VDd mice. As shown in Figure 3C, the genetic deletion of VDR reduced the mRNAs expression of *Jagged-1*, *Notch-1*, *Notch-4*, *Dll-1* and *Hes1*. Similar decreases in *Notch-1* and *Hes1* mRNAs levels were observed in VDd mice (Figure 3D). These findings indicate the positive regulatory role of VD/VDR signaling in intestinal Notch pathway.

**FIGURE 4:**
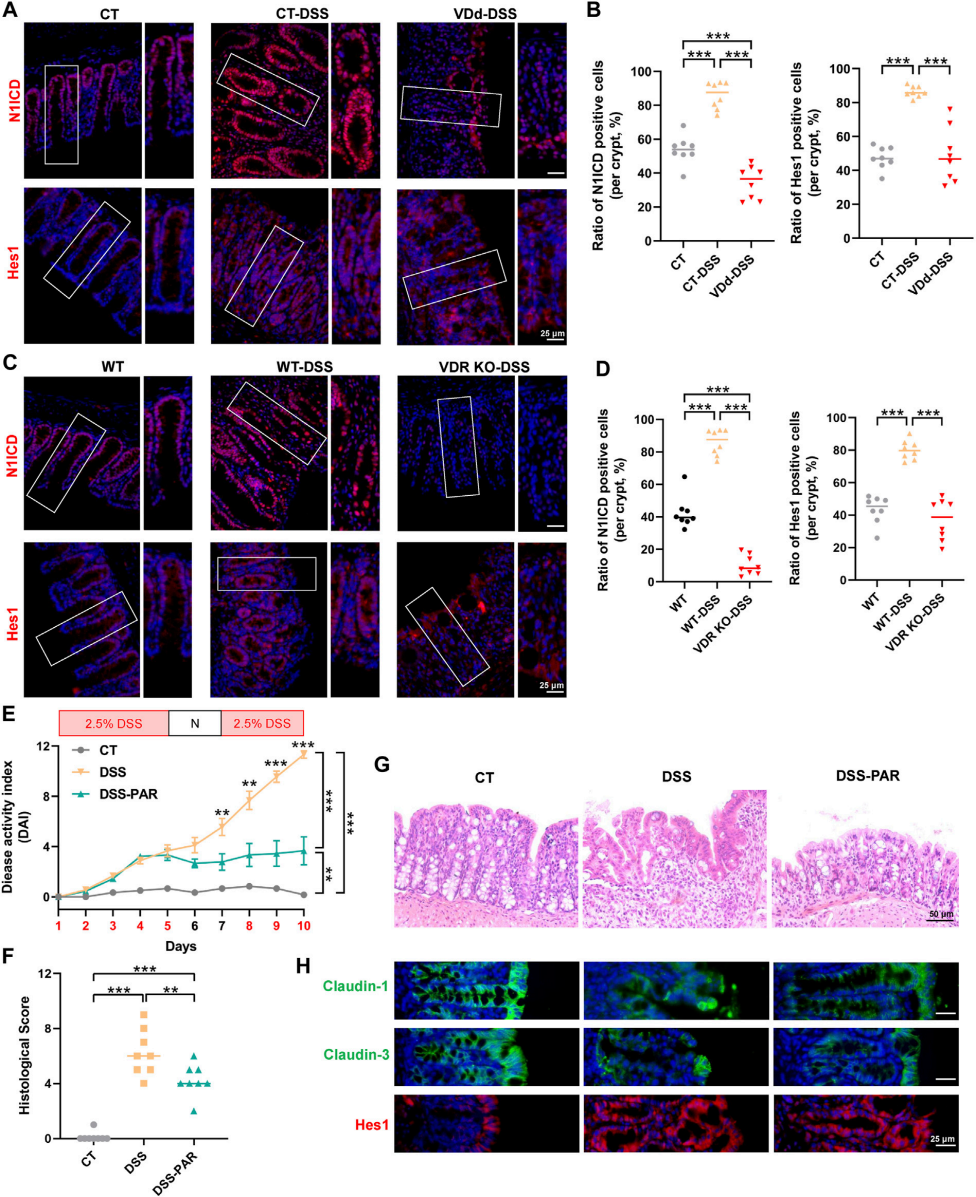
VD/VDR modulates the Notch pathway in colitis. **(A, B)** Representative colonic IF staining image and quantification of N1ICD (*red*) and Hes1 (*red*) in VDd-DSS and respective control colitis mice (scale bar = 25 μm). **(C, D)** Representative colonic IF staining image and quantification of N1ICD (*red*) and Hes1 (*red*) in VDR KO-DSS and respective control colitis mice. To activate VDR, mice were administered paricalcitol (PAR) 7 days before drinking DSS water and were sustained until sacrifice. **(E)** DAI scores of each group. DAI scores were compared by the repeated measures ANOVA with Bonferroni *post hoc* multiple comparisons. **(F)** Histological scores of distal colons in each group. Statistical analysis was performed using ANOVA with Tukey’s post-test. **(G)** Representative colonic H&E staining image of each group (scale bar = 50 μm). **(H)** Representative colonic IF staining image of claudin-1 (*green*), claudin-3 (*green*), and Hes1 (*red*) (scale bar = 25 μm). Data are presented as the mean ± SEM from 6-8 mice in each study group. *: *p* < 0.05, **: *p* < 0.01, ***: *p* < 0.001.

We further detected Notch activity in mice with DSS-induced colitis via IF staining. In VDd and VDR KO mice, DSS failed to increase nuclear translocation of N1ICD and Hes1 (Figures 4A–D). Whereas PAR treatment exhibited mucosal protection in DSS-induced colitis, which was accompanied by the decrease in DAI score and histological score in the DSS-PAR group (Figures 4E–H). Meanwhile, claudin-1 and claudin-3 protein expression and distribution partially recovered after PAR treatment, accompanied by Notch pathway activation (Figure 4H; Supplementary Figure S3).

**FIGURE 5:**
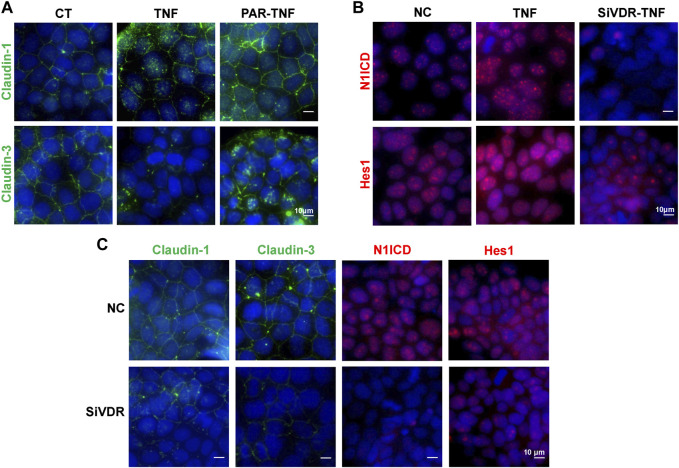
VD/VDR signaling regulates the Notch pathway and TJs in Caco-2 cells. TNF-α (100 ng/mL) was added to mimic inflammatory stimulation, and 100 nM PAR was used to activate VDR; the solvate was added as control (CT). Specific siRNA of VDR (SiVDR) was used to downregulate VDR, negative control (NC) sequence was used as control (scale bar = 10 μm). **(A)** Representative IF staining image of claudin-1 (*green*) and claudin-3 (*green*) in Caco-2 cells treated with or without PAR before TNF-α intervention. **(B)** Representative IF staining image of N1ICD (*red*) and Hes1 (*red*) in Caco-2 cells with or without VDR downregulation before TNF-α intervention. **(C)** Representative IF staining image of claudin-1 (*green*), claudin-3 (*green*), N1ICD (*red*), and Hes1 (*red*) in Caco-2 cells after VDR downregulation.

To better explore the effects of VD/VDR on the Notch pathway and TJs regulation, we used Caco-2 cells. In TNF-α-stimulated Caco-2 cells, PAR treatment suppressed claudin-1 and claudin-3 impairments that resulted from TNF-α (Figure 5A), while TNF-α failed to increase N1ICD and Hes1 expression in pre-downregulated VDR Caco-2 cells (Figure 5B). Downregulation of VDR decreased the proteins of claudin-1, claudin-3, N1ICD, and Hes1 in Caco2 cells (Figure 5C). Thus, the Notch pathway may be involved in VD/VDR-mediated TJ protection.

### 3.4 VD/VDR positively regulates Notch-1 transcription to modulate the integrity and expression of TJ

**FIGURE 6:**
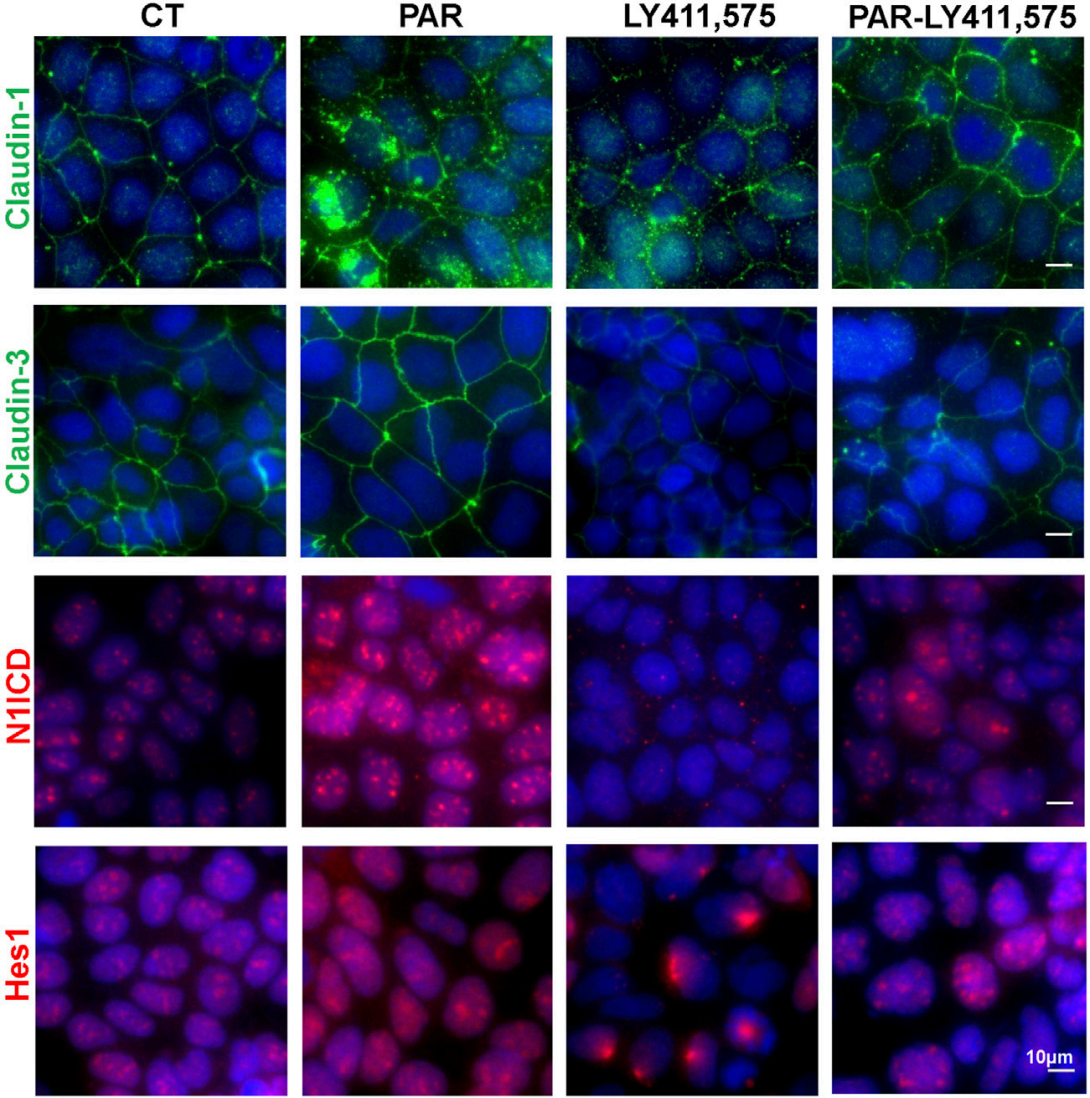
VD/VDR-mediated TJ protection is partly dependent on the Notch pathway. LY411,575 (1 μM) was used to inhibit the Notch pathway in Caco-2 cells, and 100 nM PAR was used to activate VDR. The solvate was added as control (CT). Representative IF staining image of N1ICD (*red*), Hes1 (*red*), claudin-1 (*green*), and claudin-3 (*green*) in Caco-2 cells treated with or without LY411,575 before PAR intervention (scale bar = 10 μm).

To uncover the roles of Notch pathway in VD/VDR-mediated TJ integrity and expression, we detected claudin-1 and claudin-3 via IF staining of Caco-2 cells treated with PAR and LY411,575 separately or together. Activation of the VDR by PAR treatment increased claudin-1 and claudin-3 expression as well as N1ICD and Hes1 expression and nuclear translocation (Figure 6). Inhibition of Notch by LY411,575 restricted the PAR-induced increase in N1ICD and Hes1 and recovery of claudin-1 and claudin-3 expression and distribution (Figure 6). These results suggest that VDR-mediated TJ integrity is partly dependent on the Notch pathway.

**FIGURE 7:**
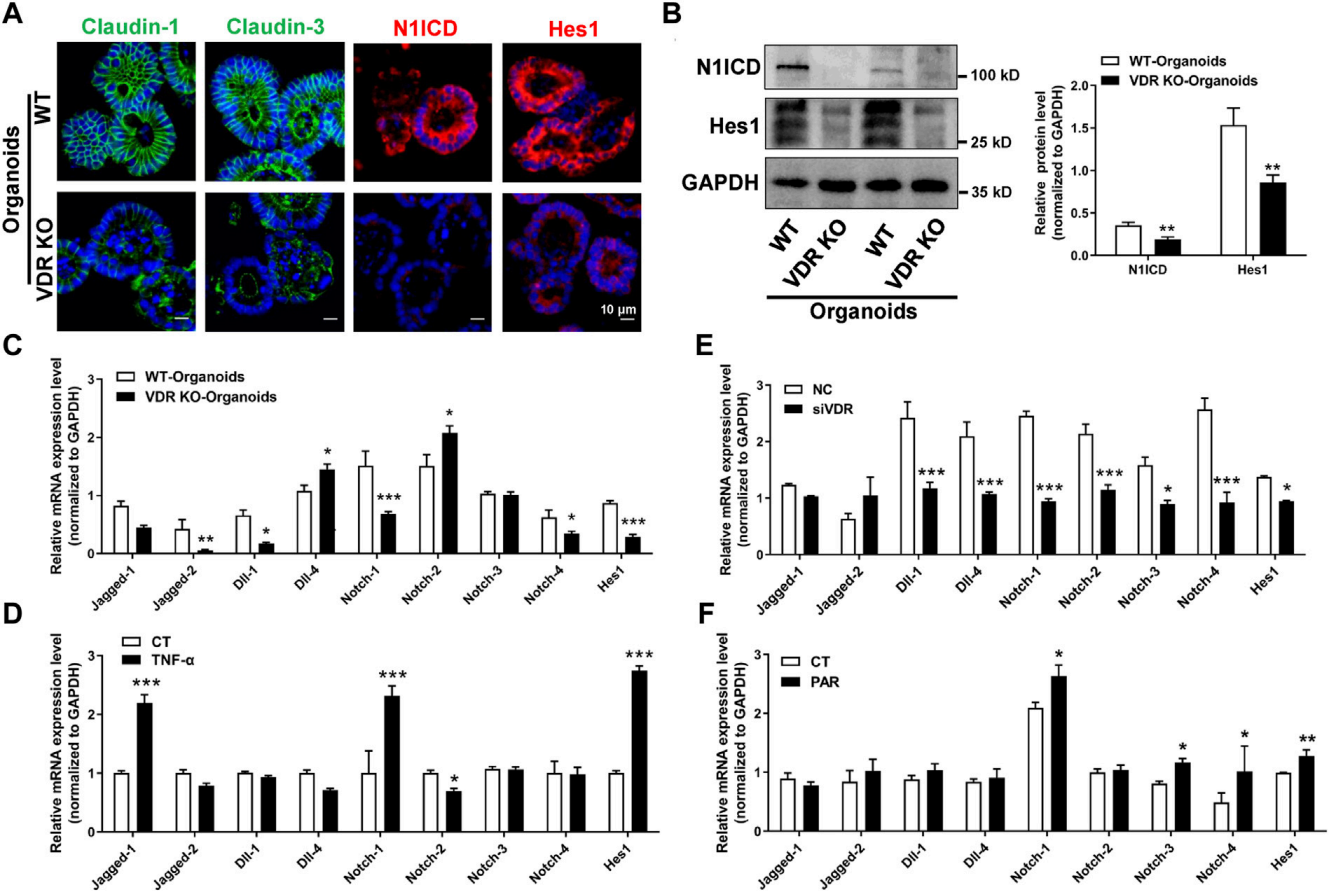
VD/VDR signaling positively regulates the Notch-1/Hes1 pathway. **(A)** Representative IF staining image of claudin-1 (*green*), claudin-3 (*green*), N1ICD (*red*), and Hes1 (*red*) in cultured intestinal organoids (scale bar = 10 μm). **(B)** Western blot analysis of N1ICD and Hes1 proteins in cultured intestinal organoids from WT and VDR KO mice. **(C)** Real-time PCR analysis of the expression of Notch ligands, receptors, and *Hes1* in cultured intestinal organoids from WT and VDR KO mice. **(D)** Real-time PCR analysis of the expression of Notch ligands, receptors, and *Hes1* in Caco-2 cells after TNF-α intervention. **(E, F)** Real-time PCR analysis of the expression of Notch ligands, receptors, and *Hes1* in Caco-2 cells after downregulation of VDR with siVDR **(E)** or activation of VDR with PAR **(F)**. Data are presented as the mean ± SEM from three separate experiments, the statistical analysis performed with paired *t*-test. *: *p* < 0.05, **: *p* < 0.01, ***: *p* < 0.001.

**FIGURE 8:**
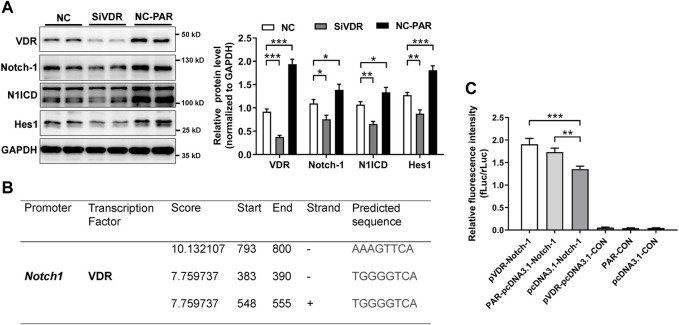
VD/VDR positively regulates Notch-1 transcription. **(A)** Western blot analysis of VDR, Notch-1, N1ICD, and Hes1 in Caco-2 cells after siVDR and PAR intervention. **(B)** Graphs of the predicted VDR binding region in the *Notch-1* promoter. **(C)** Dual-luciferase reporter assay of VDR to *Notch-1* promoter. *Notch-1* promoter (Notch-1) and negative control (CON) were constructed in a GV238 vector. The *VDR* cDNA and vector (pcDNA3.1) were linked to upregulate VDR (pVDR). Data are presented as the mean ± SEM from three separate experiments. Statistical analysis was performed using ANOVA with Tukey’s post-test. *: *p* < 0.05, **: *p* < 0.01, ***: *p* < 0.001.

To verify our findings, we used intestinal organoids from VDR KO mice. IF staining illustrated that levels of claudin-1, claudin-3, N1ICD, and Hes1 were decreased in VDR KO organoids (Figures 7A, B). Moreover, in cultured VDR KO intestinal organoids, the mRNAs expression of *Jagged-2*, *Dll-1*, *Notch-1*, *Notch-4* and *Hes1* was lower than that in WT organoids (Figure 7C). To screen potential target of VDR in the Notch pathway, we detected ligands and receptors of Notch in inflammatory stimulated and VD/VDR signaling-inhibited or -activated Caco-2 cells. We found that the mRNAs levels of *Notch-1* and *Hes1* were higher in TNF-α-stimulated Caco-2 cells (Figure 7D). In addition, we found that silencing VDR expression using its specific siRNA (SiVDR) significantly downregulated *Dll-1*, *Dll-4*, Notch receptors (1–4), and *Hes1* (Figure 7E), whereas activating VD/VDR signaling using PAR upregulated Notch receptors (1,3, and 4) and *Hes1* mRNAs (Figure 7F). Furthermore, Western blot analysis suggested that downregulation of VDR decreased Notch-1, N1ICD, and Hes1 protein levels in Caco-2 cells whereas its upregulation increased Notch-1, N1ICD, and Hes1 protein levels (Figure 8A). Taken the *in vivo* and *in vitro* results together, VD/VDR protects intestinal TJ integrity via Notch-1/N1ICD signaling.

As a transcription factor, VDR functions by binding to targeted promoter regions. We speculated that VDR might regulate Notch-1 transcription. We predicted the potential promotor regions of *Notch-1* gene where VDR might bind using an open access database ( *https://jaspar.genereg.net/* ). As shown in Figure 8B, VDR may bind to the promotor regions of *Notch-1* gene, especially in the upstream 793–800 base pairs. Results from dual-luciferase assay suggested that PAR or overexpressing VDR (pVDR) markedly elevated Notch-1 transcription (Figure 8C). These findings suggest that VD/VDR signaling positively regulates Notch-1 transcription and Notch pathway activation to modulate the integrity of TJs.

## 4 Discussion

In patients with UC and experimental colitis models, the impairment of TJs is responsible for increasing intestinal permeability, which determines disease development (Lee, 2015; Capaldo et al., 2017). In recent years, accumulating evidence has shown that VD/VDR signaling is involved in maintaining the integrity and permeability of intestinal TJs, including in UC patients (Shang and Sun, 2017; Sun and Zhang, 2022; Triantos et al., 2022). A previous longitudinal study suggested that UC patients with VD deficiency required more steroids, biologics, narcotics, emergency department visits and hospital admissions, while who received VD supplements decreased healthcare utilization (Kabbani et al., 2016). VD deficiency downregulates TJ proteins (claudin-1) (Yang et al., 2021), whereas overexpression or activation of VDR in the intestinal epithelia upregulates TJ proteins (e.g., claudin-1, claudin-5, and ZO-1) to maintain the intestinal barrier (Liu et al., 2017; Lin et al., 2019; Chatterjee et al., 2021). These findings are consistent with our results, which demonstrate the regulatory role of VD/VDR in the maintenance of TJs in the intestine. Notably, we observed compromised TJs expression, maldistribution of TJs, and increased intestinal permeability in VDd and VDR KO mice under physiological conditions. Prodromal changes in the TJ barriers indicate the potential mechanism that VD deficiency and VDR suppression are associated with increased DSS susceptibility. Based on these observations, we detected deteriorative colitis accompanied by increased intestinal permeability in DSS-induced VDd and VDR KO colitis mice, which was attributed to compromised TJs. Overall, the intestinal TJ barrier is an important target of the VD/VDR-mediated intestinal barrier protection in colitis.

Robust differentiation and proliferation of the intestinal epithelium are essential for the restoration of intestinal barrier function in chronic and relapsing inflammation (Khoramjoo et al., 2022). Notch signaling is a predominant mediator in determining the fate of differentiation and proliferation of intestinal epithelial cells, which is pivotal in maintaining intestinal architecture (Siebel and Lendahl, 2017). Hence, the role of Notch signaling in the intestinal barrier has received increasing attention. Inhibition of Notch signaling can result in spontaneous colitis (Obata et al., 2012), whereas activation of Notch signaling contributes to the reconstruction of damaged intestinal epithelium by promoting cell proliferation (Okamoto et al., 2009). Dahan et al. (2011) found that the absence of the Notch-1 signaling pathway markedly upregulated claudin-2 and downregulated occluding, and N1ICD is increased in patients with inflammatory bowel disease. Ahmed et al. (2018) showed the dysfunction of ZO-2, β-catenin, and E-cadherin in Notch suppression by dibenzazepine, which further resulted in increased permeability of epithelial cells in infectious colitis. Our present study revealed that inhibition of the Notch pathway by LY411,575 impaired the integrity of the intestinal barrier by downregulating claudin-1 and claudin-3. These findings suggest a positive regulatory effect of Notch signaling on TJ expression and intestinal barrier function.

Notably, the effect of the Notch pathway on TJs in colitis appears to be complex. The present study, along with previous works, showed an activated Notch-Hes1 pathway in TNF-α-treated Caco-2 cells or mice with DSS-induced colitis; however, the activation of the Notch pathway triggered by inflammation failed to rescue TJ injury (Okamoto et al., 2009; Wu et al., 2021). We speculated that inflammation-induced Notch pathway activation was a protective feedback mechanism that was incapable of repairing TJ impairments. However, the decompression of Notch signaling could aggravate TJs abolishment in DSS colitis. This also indicates the indispensable role of Notch activation in the protection of TJs in colitis. Interestingly, Wu et al. (2021) revealed a novel mechanism that inhibition of Notch-1/Hes1 using a compound sophorae decoction increased Muc2 secretion to alleviate colitis severity. Taken together, the results indicate that the Notch pathway is indispensable for maintaining intestinal homeostasis. However, its regulatory roles and actions in the intestinal barrier are complex and require further investigation.

Currently, the modulatory effect of VD/VDR signaling on Notch signaling is not fully understood. To understand whether Notch signaling participates in VD/VDR-mediated intestinal barrier protection, we performed a series of *in vivo* and *in vitro* experiments. First, under physiological conditions, we detected alterations in several Notch receptors, ligands, and target genes, and found that the Notch pathway was inhibited in the colons of VDd or VDR-KO mice. Moreover, the downregulation of N1ICD and Hes1 proteins were further confirmed in VD/VDR-suppressed mice or cells. Under colitis conditions, we found that VD deficiency or VDR deletion significantly suppressed colitis-induced N1ICD and Hes1 stimulation. These findings suggest the regulatory effects of VD/VDR on Notch signaling. To explore whether VD/VDR-mediated intestinal barrier protection was independent of Notch signaling, we constructed a LY411,575/PAR co-treated Caco-2 cell model. In Caco-2 cells, PAR treatment effectively stimulated claudin-1 and claudin-3 expression and upregulated and increased the nuclear translocation of N1ICD and Hes1. However, in LY411,575 pretreated Caco-2 cells, PAR treatment failed to stimulate the expression of claudin-1, claudin-3, N1ICD, and Hes1 and the nuclear translocation of the latter two. These findings indicate that VD/VDR signaling protects intestinal barrier function, which is partly dependent on the Notch pathway.

As a nuclear receptor, VDR exerts diverse effects by binding to the target promoter region and regulating transcriptional activation (Bollen et al., 2022). Therefore, VDR might regulate the transcriptional activation of the ligands or receptors of the Notch pathway. Screening of the ligands and receptors of the Notch pathway revealed that Notch-1 is closely related to VD/VDR signaling, presenting as evident and consistent alterations in various experimental models. Using a dual-luciferase reporter assay, we found that VDR directly binds to the promoter region of the *Notch-1* gene to positively regulate its transcriptional activity and further stimulate the Notch pathway. These findings uncover a potential therapeutic target of VD/VDR in the Notch signaling pathway for intestinal barrier protection.

However, there are some limitations in the present study. DSS induced colitis cannot mimic all clinical UC traits. Overexpressing N1ICD and ChIP-qPCR experiment may benefit to comprehensively reveal the relationship of VD/VDR signaling and Notch pathway in intestinal barrier protection. Besides, human colonic organoids should be used in verify our findings in the future.

In the current study, we report that VD deficiency and VDR deletion impair the intestinal mechanical barrier by downregulating TJs and promoting their abolition in mice with DSS-induced colitis. VD/VDR signaling, which regulates TJ protection, is dependent on the Notch pathway, and VDR promotes Notch-1 transcription by directly binding to its promoter. In conclusion, VD supplementation (or VDR activation) maintains TJ integrity and the intestinal barrier against colitis by positively regulating the Notch pathway, thus providing a promising therapeutic strategy for intestinal mucosal restoration in UC.

## Data Availability

The original contributions presented in the study are included in the article/Supplementary Material, further inquiries can be directed to the corresponding authors.

## Supplementary material

SUPPLEMENTARY FIGURE S1 Illustration and weight alteration of animal experiments. **(A)** Colitis was induced in VDd mice using 2.5% DSS water. **(B)** Colitis was induced in VDR KO mice using 2.5% DSS water. **(C)** DSS-induced colitis modeling with Notch inhibition. Inhibition of Notch signaling was induced via gavage of 10 mg/kg LY411,575 in the last 4 days. **(D)** DSS-induced colitis modeling via paricalcitol (PAR) treatment. Mice were intraperitoneally administered 300 ng/kg PAR once per day. **(E)** Serum concentration of 25(OH) VD_3_ in mice.

SUPPLEMENTARY FIGURE S2 Original images and quantification of immunofluorescence staining for claudin-1 and claudin-3. Quantification was determined by the fluorescent integrated density (IntDensity) per high power field (HPF) by ImageJ software. **(A)** Colonic IF staining image of claudin-1 (*green*), claudin-3 (*green*), and the nucleus (stained with DAPI, *blue*) as shown in Figure 1E, and graphs for quantitative analysis. **(B)** Colonic IF staining image of claudin-1 (*green*) and claudin-3 (*green*) as shown in Figure 1J, and graphs for quantitative analysis. **(C)** Colonic IF staining image of claudin-1 (*green*) and claudin-3 (*green*) as shown in Figure 2E, and graphs for quantitative analysis.

SUPPLEMENTARY FIGURE S3 Colonic IF staining image of claudin-1 (*green*), claudin-3 (*green*) and Hes1 (*red*) as shown in Figure 4H, and graphs for quantitative analysis.
