# Supplementary figures and images for: Vitamin D/vitamin D receptor protects intestinal barrier against colitis by positively regulating Notch pathway
Source: Front Pharmacol. 2024 Jul 26;15:1421577. doi: 10.3389/fphar.2024.1421577 (PMC11310051; doi:10.3389/fphar.2024.1421577)

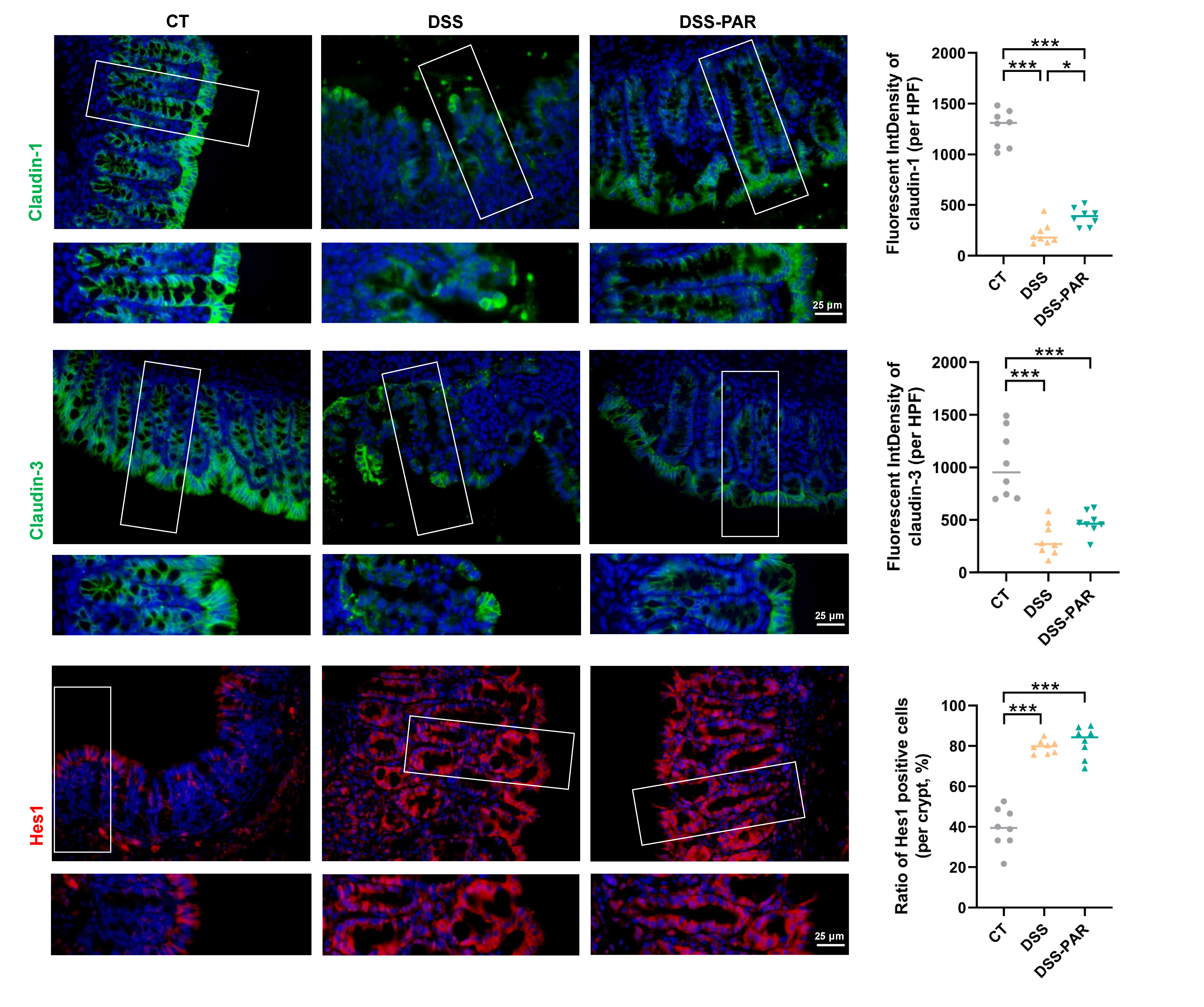

Supplement: Supplementary file 1 [file Image3.JPEG]

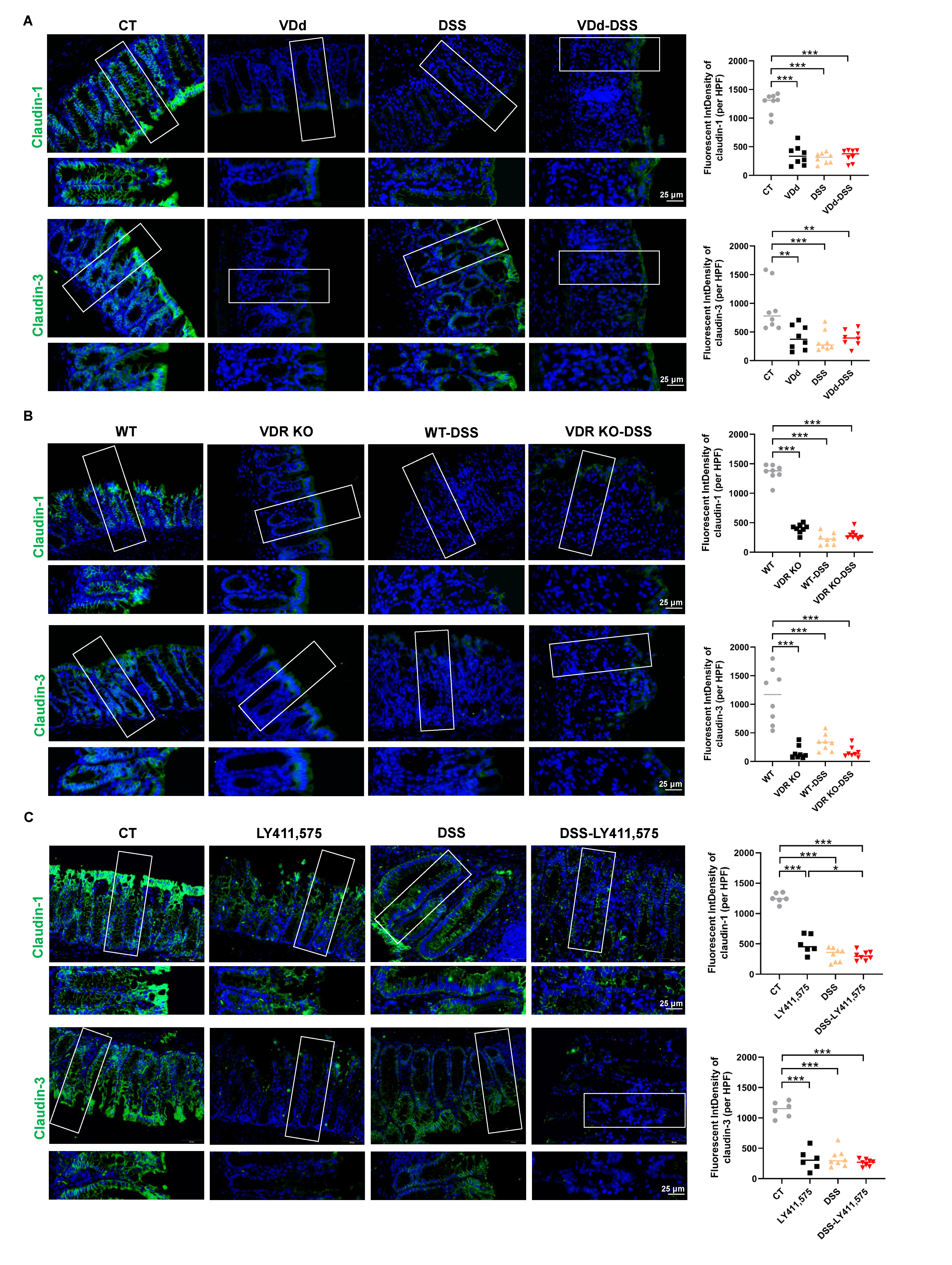

Supplement: Supplementary file 2 [file Image2.JPEG]

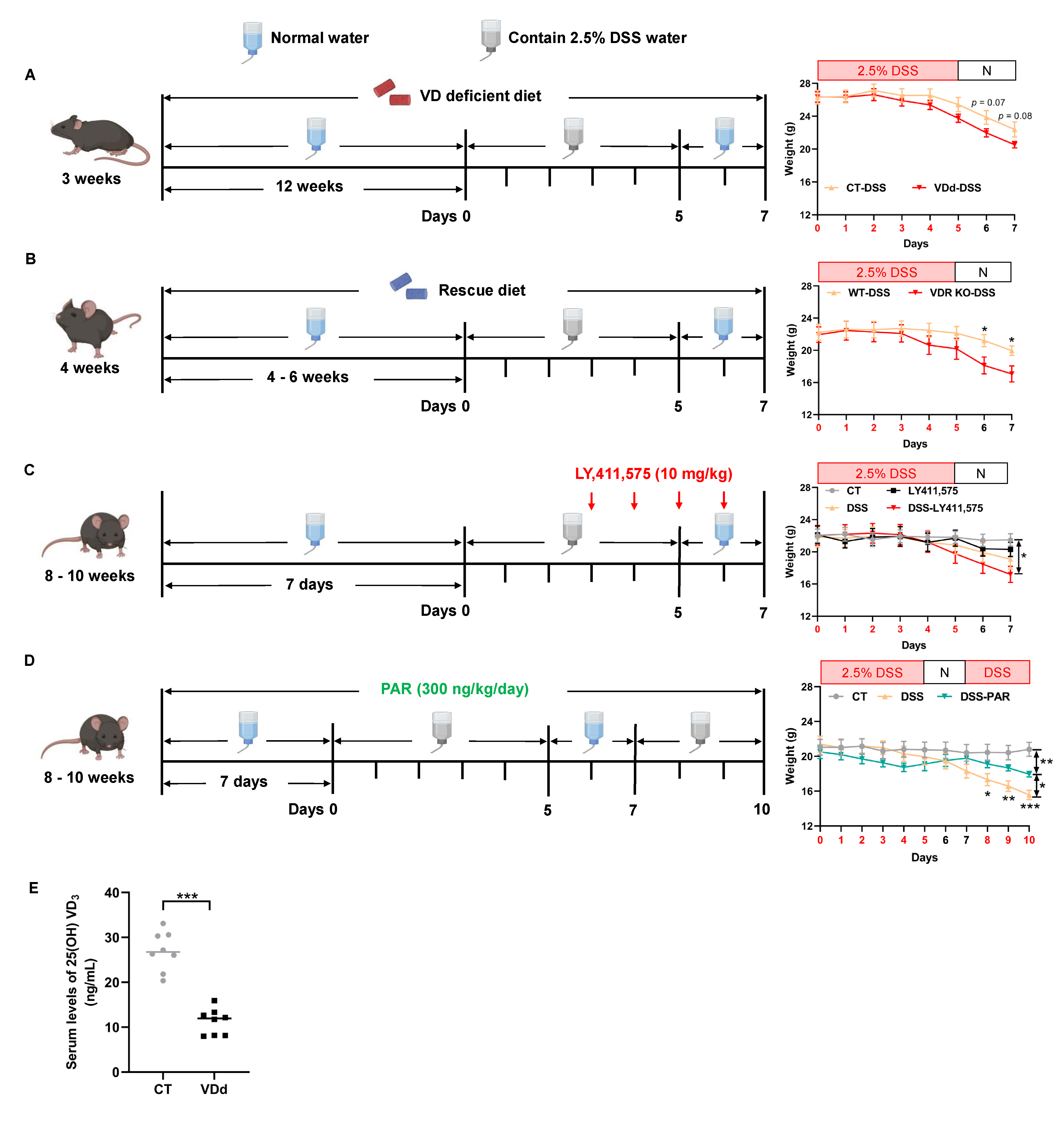

Supplement: Supplementary file 3 [file Image1.jpg]
